# The REST (randomised evaluation of sleeping with a toy or comfort item) trial: a protocol for an online, randomised trial of comfort item use on sleep quality in children

**DOI:** 10.1016/j.conctc.2025.101580

**Published:** 2025-11-25

**Authors:** Simone Lepage, Laura Flight, Nikki Totton, Declan Devane

**Affiliations:** a School of Nursing and Midwifery, University of Galway, 26 Upper Newcastle, Galway, H91 E3YV, Ireland; b Health Research Board–Trials Methodology Research Network (HRB-TMRN), University of Galway, 26 Upper Newcastle, Galway, H91 E3YV, Ireland; c National Institute for Health and Care Excellence, 3rd Floor, 3 Piccadilly Place, Manchester, M1 3BN, United Kingdom; d Sheffield Centre for Health and Related Research (SCHARR), School of Medicine and Population Health, University of Sheffield, 30 Regent St, Sheffield City Centre, Sheffield, S1 4DA, United Kingdom; e Evidence Synthesis Ireland, University of Galway, 26 Upper Newcastle, Galway, H91 E3YV, Ireland; f Cochrane Ireland, University of Galway, 26 Upper Newcastle, Galway, H91 E3YV, Ireland

**Keywords:** Protocol, Randomised trial, Citizen-science, Patient and public involvement, Children, Sleep quality, Online trial

## Abstract

Randomised controlled trials (RCTs) are the gold standard for evaluating healthcare interventions. Participatory research, in which the public is engaged in research activities, enhances their understanding of trials but requires innovative strategies to reach diverse populations, particularly children. This article outlines the design of the REST (Randomised Evaluation of Sleeping with a Toy or comfort item) trial, a child-led study investigating whether sleeping with a comfort item affects sleep quality in children compared to not using one.

The REST trial was created with children aged 7 to 12 through The Kid's Trial, an online initiative where children co-design and co-conduct a randomised trial. The REST trial is a two-arm, pragmatic, superiority RCT. Children worldwide participate from home and are randomly assigned (1:1) to either sleep with or without a comfort item for 7 nights. The primary outcome is sleep-related impairment (SRI), measured using the PROMIS Pediatric Short Form v1.0 Sleep-Related Impairment 4a questionnaire. The secondary outcome is sleep quality, evaluated using a single-item Sleep Quality Scale. Data are collected via online self-reported questionnaires at baseline and eight days post-randomisation. Recruitment is global, targeting caregivers through online media, with study materials available on a dedicated website.

The REST trial aims to enrol 292 participants to achieve 80 % power to detect a 3-point difference in SRI. Findings will explore the impact of comfort items on sleep and demonstrate the feasibility and benefits of child-led participatory research, fostering scientific literacy and critical thinking.

## Highlights

• This protocol describes the design and rationale of an RCT co-created with children.
• The REST trial question and methodology were determined by children aged 7 to 12.
• The REST trial was co-designed with kids participating in The Kid's Trial.
• The REST trial is a unique example of a global, online, participatory trial with children.

**Table:** Glossary

RCT	Randomised Controlled Trial
PROMIS	Patient Reported Outcomes Measurement Information System
SRI	Sleep-Related Impairment
SQS	Sleep Quality Scale
SAP	Statistical Analysis Plan
MID	Minimally Important Difference
CI	Confidence Interval
SD	Standard Deviation
ANCOVA	Analysis of Covariance
GDPR	General Data Protection Regulation Act
DPO	Data Protection Office
DPIA	Data Protection Impact Assessment
PP	Per Protocol

## 1 Introduction

Randomised controlled trials are designed to minimise bias when evaluating healthcare interventions and can provide evidence to researchers to determine the effectiveness of an intervention [1]. There is evidence that when the public understands the processes and importance of randomised trials, they are more likely to provide input and participate in them, but we know this understanding is lacking [2]. To educate the public on the value and methods of trials, researchers need to find creative ways to engage them. Developing ways to reach a large audience for educational purposes can be challenging. However, in 2019, a group of researchers devised an innovative way to do this by conducting The People's Trial, an online project that educated adults about randomised trials [3]. The People's Trial engaged members of the public in the design and conduct of their own randomised trial, resulting in over 3000 people from 72 countries taking part [3].

Our research project, The Kid's Trial, was inspired by, builds on, and is run similarly to The People's Trial. The Kid's Trial, like The People's Trial, occurs entirely online. When a trial has some or all of its methods occurring away from a central point of conduct, it is called a decentralised trial [4]. Decentralised methods may include collecting data via mobile clinics rather than a single hospital, using digital technologies (e.g., wearable devices), or moving a trial online [4]. Decentralised trial methods can reduce participants' burden of involvement [5], offer broader recruitment opportunities [6], and reduce research waste [[5], [6], [7]].

The Kid's Trial allows primary school-aged children (between 7 and 12 years of age) to participate in all the steps involved in designing, conducting, and reporting a randomised trial in a safe and fun way. The objective of the project is to help children understand randomised trials and why they matter. We believe this will increase and encourage children's critical thinking, especially regarding health claims they encounter daily.

The Kid's Trial opened in March of 2024, and in the first step, children across the globe were invited to submit low-risk, engaging, health-related questions they wished to investigate. Subsequently, participants voted to select their favourite question and collaboratively designed the methodology to address it. The selected research question: *Does sleeping with a comfort item (such as a soft toy or special blanket) make a difference in how well kids sleep compared to not sleeping with a comfort item?* forms the basis of this study. This innovative, child-led randomised trial, designated the REST (Randomised Evaluation of Sleeping with a Toy or comfort item), will recruit children to participate and report their results. The REST trial is a two-arm, parallel, superiority trial that will allocate participants in a 1:1 ratio to either sleep with or without a comfort item for seven nights.

## 2 Methods

### 2.1 Study design

This study was prospectively registered on January 10th, 2025, with ISRCTN, ISRCTN13756306, https://doi.org/10.1186/ISRCTN13756306, and received ethical approval on January 16th, 2023, by the University of Galway Research Ethics Committee (Ref: 2023.02.014). The REST trial began recruitment on January 13th, 2025 and is ongoing at the time of this manuscript's preparation. This protocol was developed using the SPIRIT reporting guidelines (Standard Protocol Items: Recommendations for Interventional Trials) [8,9]. The SPIRIT and SPIRIT-Outcomes checklists are available in Appendix A.1. This is a two-arm, online, pragmatic, superiority, randomised controlled trial (RCT). The REST trial's research question emerged from the first step of The Kid's Trial, called 'Choosing the Question!' Children submitted potential research questions, which were then refined through two rounds of voting by the participating children. Before the final voting round, we searched the Cochrane database, the University of Galway library database, and Google Scholar to confirm that randomised trials had not previously addressed the top three candidate questions. The objective of the study is to determine whether sleeping with a comfort item for seven days affects children's sleep compared to not sleeping with a comfort item for seven days. Children from around the world can enrol in the trial and will be randomly assigned to either the intervention group (sleeping with a comfort item) or the control group (sleeping without a comfort item). They will conduct their trial at home for seven days.

### 2.2 Study setting

All participants will participate from their own homes. The trial is open globally, and any child who can access the website is invited to join. All trial materials are available online at https://www.thekidstrial.ie/.

### 2.3 Participants

All participants are self-referred. Participants are primary school-aged children (7–12 years old) who meet the inclusion criteria. Inclusion criteria are confirmed via guardian consent and participant assent using QuestionPro Software Surveys [10]. Guardian consent and participant assent are obtained via QuestionPro [10] in digital form, as approved by our Research Ethics Committee. The Kid's Trial, and in turn, the REST trial, is open to any child worldwide, meaning there are no geographical exclusion criteria. However, due to budgetary constraints, we could not translate all trial materials into other languages; thus, a good understanding of English is required. Inclusion and exclusion criteria are listed below.

#### 2.3.1 Inclusion criteria

• Children aged 7–12 years.
• Proficiency in English sufficient to understand trial materials.
• Access to the trial's online platform.
• Guardian consent for participation.

#### 2.3.2 Exclusion criteria

• Inability to understand and provide assent.

#### 2.3.3 Participant characteristics and demographics

Demographic information collected includes a guardian's email address, as well as the age, gender, country of residence, and ethnicity of the participating child. Because of the age of participants, demographic information is entered by a consenting guardian. Guardians are only required to provide a participant's age and a guardian's email address. However, we request gender, country of residence, and ethnicity to help determine the trial's inclusivity and geographical reach.

### 2.4 Interventions

#### 2.4.1 Intervention: sleeping with a comfort item

For seven days, participants in the intervention, also called the **‘Try-It-Out’ group, are asked to:**

• Select a toy or comfort item and sleep with it nightly.
• Use the same toy or comfort item throughout the trial.
• Begin using the comfort item during bedtime routines.
• Sleep in their usual bed.
• Maintain usual bedtime habits while adding the comfort item.

The comfort item used by children can be a toy, blanket, item of clothing, or any item they decide might bring them comfort. Bedtime routines are defined as anything children do each night to prepare for bed; for example, if they read each night before bed, they are instructed to start using their comfort item then. We define ‘usual bed’ as a bed that the child sleeps in regularly, meaning that if they have more than one family home or live at a boarding school, these would also be considered their ‘usual bed’.

#### 2.4.2 Control: sleeping without a comfort item

For seven days, participants in the control group, also known as the ‘Wait-and-See’ group, are asked to.

• Avoid the use of any comfort items during the trial period.
• Sleep in their usual bed.
• Maintain usual bedtime habits.

Children in both groups are asked about their usual use of a comfort item at night at baseline. If children in the control group usually use a comfort item, they are asked not to use one for the seven nights of the trial. For this group, the same definition of ‘usual bed’ is used. Otherwise, no changes should be made to their usual bedtime habits and routine. During the trial, children in both groups are asked to pay attention to their daytime sleepiness and overall sleep quality.

### 2.5 Outcome measures

**Fig. 1:**
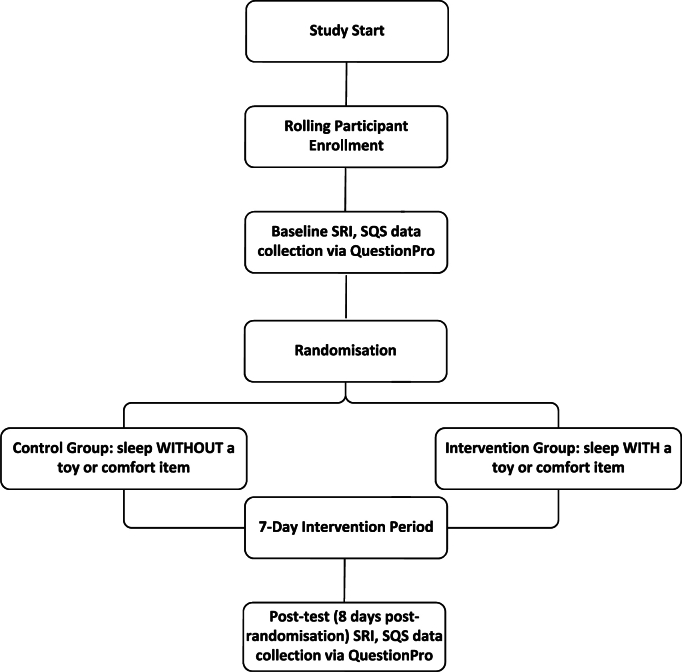
Participant flow diagram for the REST trial.

Outcome measures are collected via self-reported questionnaires, assessed at baseline and 8 days post-randomisation. Fig. 1 shows the flow diagram for a participant's timeline. Baseline questionnaires are administered using QuestionPro Survey Software [10] via a link on The Kid's Trial website when a child enrols in the trial. Post-trial measurement questionnaires are administered via a link in QuestionPro Survey Software [10] on day eight post-randomisation, emailed to participants' guardians. To enhance retention and reduce missing data, guardians will receive two email reminders if children have not completed their questionnaires by day 10 post-randomisation. Email reminders will be sent on day 10 and day 13 post-randomisation.

#### 2.5.1 Primary outcome

The children who participated in previous steps of The Kid's Trial chose the question they wanted the trial to answer and how they wanted to measure “a difference to how well kids sleep”. Daytime sleepiness was ranked as the number one indicator of whether children were sleeping well by the kids who participated in the ‘Planning the Trial!’ step.

The primary outcome of daytime sleep-related impairment (SRI) will be measured using the Patient Reported Outcomes Measurement Information System (PROMIS) Pediatric Short Form v1.0 Sleep-Related Impairment 4a [11]. Participants will complete this questionnaire at baseline and post-trial. The questionnaire consists of four questions about sleepiness during usual waking hours. The instrument has four short questions about daytime sleep-related impairment and asks the participant to choose one of five categorical answers: Never, Almost Never, Sometimes, Almost Always, Always. The lowest possible score is 4 points, and the highest is 19. Each question is scored according to the scoring manual, which includes a conversion table to score each answer to a T-score [12]. Further details for scoring these questionnaires are available in the statistical analysis plan (SAP) in Appendix A.2. Primary and secondary outcome questionnaires are available in Appendix A.3.

#### 2.5.2 Secondary outcome & covariates

The secondary outcome is sleep quality, measured using the Single-Item Sleep Quality Scale (SQS) [13] at baseline and post-trial. The SQS consists of one question asking participants to rate their overall sleep quality over the previous seven days on a visual scale from 0 to 10, with 1-unit increments, and five possible answers. The options are categorical, where 0 is terrible, 1–3 is poor, 4–6 is fair, 7–9 is good, and 10 is excellent. Therefore, the highest score (excellent or 10) is scored as a 5, and the lowest (terrible or 0) is scored as a 1. No further conversion is needed to score this measure.

The fixed covariates are baseline measurement of the outcome being analysed, baseline comfort item use, age, and gender. The country of residence will serve as the random effect, as detailed in the ‘Sample Size Calculation' section and in our SAP (available in Appendix A.2). All questionnaires are accessible in Appendix A.3.

#### 2.5.3 Adherence and treatment integrity

Participants are asked post-trial, whether they followed their group's instructions, using Likert scales ranging from Never (1–2 nights, 3–4 nights, 5–6 nights) to Always. If the participant's response is 3–4 nights or less frequently, they will be considered non-adherent and asked why they deviated from their group's instructions. This customised questionnaire is available in Appendix A.3.

### 2.6 Sample size calculation

The details of the sample size calculations are available in the SAP (A.2). The calculation is based on a two-sided superiority test to determine if sleeping with a toy or comfort item makes a difference to primary-school-aged children compared to not sleeping with a toy or comfort item using the PROMIS Pediatric Short Form v1.0 Sleep-Related Impairment 4a [11]. The sample size calculation is based on a precedent that allows for the use of the repeated measures of the baseline and day eight post-randomisation for both instruments (PROMIS SRI and SQS) in a linear mixed effects model with an Analysis of Covariance (ANCOVA) formula, using a correlation between the repeated measures of 0.5 [14]. For the primary outcome, PROMIS SRI, we assume a minimally important difference (MID) of 3 points [15], a standard deviation (SD) of 10 [16], and a dropout rate of 10 % [17].

With a sample size of 131 participants per group, a two-sided test with a 0.05 significance level has 80 % power to detect a minimally important difference in SRI with two time point measurements when the SD is 10, the correlation coefficient is 0.5. When using an estimated dropout rate of 10 %, the group sizes increase to 146 participants per group or 292 participants overall.

If the target sample size is reached before the planned closing date, enrolment will remain open to allow as many children as possible to participate. The closing date will be determined by time and resource limitations rather than statistical considerations. While most clinical trials would stop recruitment once the target is met for ethical and methodological reasons, this trial is unique in that it is embedded within a broader citizen-science study designed to help children understand what RCTs are, why they matter, and how they support critical thinking. Restricting enrolment would exclude interested children from a valuable learning opportunity, thereby contradicting the study's educational and participatory goals. Our statistical analysis plan includes sensitivity analyses that will allow us to interpret effect sizes appropriately, regardless of the final sample size achieved.

### 2.7 Recruitment

Children worldwide are eligible to participate, and the main recruitment avenues are traditional and social media. Most social media platforms have a minimum age of 13, so social media recruitment targets only caregivers. Recruitment materials are being disseminated within our personal and professional networks, and in countries where we have no established networks, we are working with children's advocacy groups we have identified to aid in our recruitment efforts. Recruitment is passive and requires children and their caregivers to initiate engagement with the trial via email, social media, or through the trial's dedicated website: https://www.thekidstrial.ie/. The website contains all pertinent data and privacy information, as well as the Participant Information in the form of the ‘Parents’ Informational Flipbook’ and the ‘Children's Information Flipbook’ found on the homepage. Children and their parents self-select to join the REST trial by navigating through the website to the ‘Running the trial!’ page and clicking a ‘Join the trial HERE’ button. Participants are then redirected to a QuestionPro Software Survey [10] and randomised to their allocated group, either the intervention or control group.

### 2.8 Randomisation and blinding

Assenting, eligible participants who have received guardian consent fill out baseline measurement questions upon clicking the ‘Join the trial here!’ button on the ‘Running the Trial!’ page on the website. Following these questions, participants are randomised to either the intervention or control group. Random group allocation to either the intervention or the control group is computer-generated using simple randomisation through the QuestionPro Survey Software [10]. Participants are randomised with equal probability (1:1 ratio), but they cannot predict which group they will be allocated to, as enrolment is rolling and individualised. The participants and the researchers become aware of the allocation after randomisation, but neither can influence it. Due to resource constraints, it is not possible to blind researchers to participants' group allocation, and because of the nature of the intervention, it is impossible to blind participants to group allocation.

Participants start their trial the day after they are randomised to their treatment group. Eight days after joining the trial, participants’ guardians are sent an email link to the QuestionPro [10] survey unique to their allocated group (either intervention or control). Post-trial, participants will answer the same questions they were asked at baseline, with the addition of group-specific intervention fidelity questions (available in Appendix A.3).

### 2.9 Data management

#### 2.9.1 Data collection

All data is collected directly in QuestionPro [10] surveys. Once surveys are complete, raw data will be downloaded, and duplicates removed. Each participant will receive a unique numeric identifier (pseudonymisation), or if they have taken part in previous steps of The Kid's Trial, they receive the same unique identifier as in previous steps. There is no independent data management committee, as the trial addresses a low-risk question.

#### 2.9.2 Analysis

The REST trial is a two-arm, parallel, pragmatic, superiority trial and will be reported and presented in accordance with the updated CONSORT [20] statement for parallel-group randomised trials. The statistical analyses will be performed on an intention-to-treat basis. A Per Protocol (PP) analysis will also be conducted as a sensitivity analysis to assess the robustness of findings. All statistical exploratory tests will be two-sided with a 0.05 significance level. Baseline demographics, comfort item use, sleep-related impairment (PROMIS Pediatric Short Form v1.0 SRI 4a), and overall sleep quality (SQS) will be described and summarised overall, and for both treatment groups.

The primary aim is to compare the sleep of children who used a toy or comfort item at night with that of children who did not. The primary outcome is a participant-reported outcome using the PROMIS SRI, instrument [11]. We will use a linear mixed effects model to analyse this data. In our analysis, the treatment group serves as a predictor, and the fixed covariates are baseline measurement of the outcome being analysed, baseline comfort item use, age, and gender. The country of residence will serve as the random effect. Secondary outcomes will be analysed similarly. A 95 % confidence interval (CI) for the mean difference in this parameter between the intervention (comfort item) and control (no comfort item) groups will be calculated. Multiple imputation techniques will be considered if the primary outcome has missing data exceeding 10 %. For further details, please see the SAP (available in Appendix A.2).

#### 2.9.3 Handling and storage of data

We will collect informed assent and guardian consent for each participant. Demographic information will be entered by the consenting adult. Participant data will be handled in accordance with the EU General Data Protection Regulation (GDPR) and University policies. All data collected during the study will be stored securely on a University of Galway cloud-based server in accordance with the University of Galway Research Data Management Policies. Participants’ identities will remain anonymous throughout the study; participants will not interact, and all collected data will be pseudonymised, encrypted, and password-protected as per university guidelines. Pseudonymisation keys are password-protected, encrypted, and stored separately from the data. Access to the data and pseudonymisation keys will be restricted to the principal investigator (SL) and her PhD supervisor (DD).

After analysis, the pseudonymised data will be anonymised. After seven years, per the University of Galway Data Retention Policy, all data will be destroyed in a confidential manner. A Data Protection Impact Assessment (DPIA) was filed with the University of Galway's Data Protection Office (DPO) at the time of the ethics review. Analysed, anonymised data will be publicly available and stored in the Open Science Framework repository [18].

#### 2.9.4 Adverse events/data breaches

We do not anticipate any emotional or physical risks or disturbances to participants or their guardians in the REST trial. The trial question, "Does sleeping with a comfort item, for example, a soft toy or special blanket, make a difference to how well kids sleep compared with not sleeping with a comfort item?" is a low-risk intervention that reduces the possibility of any harm associated with the trial. All participants and their guardians are provided with contact information for the research team, the University of Galway's Research Ethics Committee (REC), and DPO in case of any questions or concerns. Participants are also informed on the website where and how to make a complaint to the Irish Data Protection Commission. Should any participant disclose current or historical abuse, the research team will appropriately escalate the disclosure in accordance with the Children First Act of 2015 [19]. Should any disclosure be made by a child outside of Ireland, we will endeavour to identify reporting mechanisms in the country where the child lives.

#### 2.9.5 Trial ethics, registration, consent, and assent

The University of Galway REC, ref. no. 2023.02.014, approved this study. The trial registration will be updated in the case of changes to the study protocol. Informed consent and informed assent (digitally collected via QuestionPro surveys) will be collected for all participating children and one of their guardians. The University of Galway REC will be notified of any adverse events that occur with participants of this study. The University of Galway DPO will be notified of any data-handling or storage breaches in accordance with their policies.

The website's Privacy Notice, the Parents' Information Flipbook, and the Children's Information Flipbook provide a plain, age-appropriate explanation of why we are conducting this study and our data handling protocol.

Participants may withdraw from the study at any time without notice, explanation, or consequence to themselves or their guardians. They are made aware of this during the consent and assent process and in their ‘Information Flipbooks’.

## 3 Discussion

The REST trial emerged from The Kid's Trial, an innovative participatory research project in which children aged 7–12 design and conduct their own randomised trial. This unique approach means that many young participants in the REST trial helped conceive and plan it. Through The Kid's Trial, children gain valuable exposure to the principles of randomised trials and develop critical thinking skills when evaluating the health claims they encounter daily. While full participation in The Kid's Trial's activities, meaning all the steps from choosing a question through sharing the trial's results, provides the richest learning experience, even those who only join the REST trial will develop a better understanding of scientific research while contributing to our knowledge of children's sleep patterns.

The REST trial uniquely explores the impact of comfort items on children's sleep quality—a relevant topic worldwide, as evidence shows that children across age groups and cultures experience insufficient or disrupted sleep, which has direct implications for their well-being [[20], [21], [22]]. Sleep is a critical component of health and development [22,23], and understanding modifiable factors, such as comfort items, may offer practical insights for caregivers and healthcare providers. The trial's decentralised design broadens its reach, enabling participation from diverse cultural and geographic backgrounds. This inclusivity not only enhances the external validity of the findings but also enables the exploration of cross-cultural differences in sleep behaviours and comfort item use, as sleep patterns and habits vary significantly across geographic and cultural backgrounds [24].

### 3.1 Strengths and limitations

Several features distinguish the REST trial from traditional randomised controlled trials. The REST trial represents an inclusive and participatory research approach by inviting children to contribute to its design. The trial's global, online nature minimises logistical barriers, enabling participation from a wide range of settings. This approach also reduces the burden on participants, making it feasible for families to engage without significantly disrupting their routines. Beyond generating data, the trial emphasises education, making scientific concepts accessible to children and their families through engaging and age-appropriate materials.

Despite its strengths, the REST trial has the following limitations. Although the trial aims to be global, materials are only available in English, excluding non-English speaking populations. The seven-day intervention period may not capture long-term effects or variability in sleep patterns due to external factors, such as environmental influences or family routines. However, a shorter trial is justified to engage with and facilitate children's participation. This ensures that the trial is not disruptive and allows us to reach a large sample of children, increasing their scientific literacy and awareness of randomised trials. Participants and researchers are aware of group allocation, which could introduce bias. While the trial design allows for a pragmatic mix of children with varying baseline comfort-item use, this unconventional approach, though strengthening external validity, means that not all participants will experience a change in their routine when randomised. We have addressed this through covariate adjustment and planned subgroup analyses. We acknowledge that, although this trial's online nature makes it generally more accessible to children and their guardians, a digital divide may exclude some families from participating**.**

### 3.2 Conclusion

The REST trial highlights the potential of decentralised citizen-science research to involve and educate children while advancing our understanding of paediatric sleep. By focusing on inclusivity, accessibility, and education, the trial addresses an important research question and serves as a model for engaging children in scientific research. Future iterations of such citizen-science trials could incorporate insights from the behaviour-change literature to examine not only the effects of adopting new health behaviours but also the challenges and benefits of discontinuing established habits. The results aim to inform future interventions to improve children's sleep and highlight the value of participatory research in promoting scientific literacy among young people.

## CRediT authorship contribution statement

**Simone Lepage:** Writing – review & editing, Writing – original draft, Visualization, Validation, Resources, Project administration, Methodology, Investigation, Formal analysis, Data curation, Conceptualization. **Laura Flight:** Writing – review & editing, Visualization, Validation, Supervision, Resources, Project administration, Methodology, Investigation, Formal analysis, Conceptualization. **Nikki Totton:** Writing – review & editing, Visualization, Validation, Supervision, Formal analysis. **Declan Devane:** Writing – review & editing, Writing – original draft, Visualization, Validation, Supervision, Resources, Project administration, Methodology, Investigation, Funding acquisition, Formal analysis, Data curation, Conceptualization.

## Ethics

This study received ethical approval on January 16, 2023, Ref: 2023.02.014, from the University of Galway Research Ethics Committee, The Research Office, University of Galway, Galway, H91 TK33, Ireland.

## 4 Trial status

Participant recruitment started in January 2025 with version 1.0 of this protocol. At the time of manuscript preparation, 93 participants were enrolled. This trial was prospectively registered on January 10, 2025 (ISRCTN13756306; https://doi.org/10.1186/ISRCTN13756306).

## Funding sources

This research is funded by the Health Research Board of Ireland [HRB-TMRN-2021-001]. This work was also supported by the College of Medicine, Nursing and Health Sciences, University of Galway, Ireland. DD is the grant holder. The funders had no role in designing this study and will not have any role in its execution, analyses, data interpretation, or decision to submit results.

## Declaration of competing interest

No competing interests were disclosed.

## Data Availability

No data was used for the research described in the article.

## Appendices

Open Science Framework (OSF) repository: The REST (Randomised Evaluation of Sleeping with a Toy or comfort item) trial: https://doi.org/10.17605/OSF.IO/WU7Q3 [18]. **All data are available under the terms of the Creative Commons Zero “No rights reserved” data waiver, CC0 1.0 Public domain dedication.**

The project contains the following underlying data:
