# Supplementary material for: The REST (randomised evaluation of sleeping with a toy or comfort item) trial: a protocol for an online, randomised trial of comfort item use on sleep quality in children
Source: Contemp Clin Trials Commun. 2025 Nov 25;48:101580. doi: 10.1016/j.conctc.2025.101580 (PMC12702047; doi:10.1016/j.conctc.2025.101580)
Supplement: Supplementary file 1 — Multimedia component 1 [file mmc1.pdf]

Rest Trial Extended Data (ED.1): WHO Data Set

WHO Data Set

Primary Registry and Trial Identifying Number: ISRCTN13756306

Date of Registration in Primary Registry: 10 January, 2025

Secondary Identifying Numbers: N/A

Source(s) of Monetary or Material Support: Health Research Board—Trials Methodology Research Network in Ireland (grant ref: HRB-TMRN-2021-001) and the College of Medicine, Nursing and Health Sciences, University of Galway, Ireland.

Primary Sponsor: University of Galway College of Medicine, Nursing, and Health Sciences, University of Galway, Ireland

Contact for Public Queries: Simone Lepage, PhD Candidate, BSc, MSc, BSN, RGN, RM
School of Nursing and Midwifery
Aras Moyola
University of Galway, H91 HX31
Galway, Ireland
T: (091) 493 432
E: s.lepage1@universityofgalway.ie

Contact for Scientific Queries: Simone Lepage, Principal Investigator, PhD Candidate, BSc, MSc, BSN, RGN, RM
School of Nursing and Midwifery
Aras Moyola
University of Galway, H91 HX31
Galway, Ireland
T: (091) 493 432
E: s.lepage1@universityofgalway.ie

Public Title: The REST (Randomised Evaluation of Sleeping with a Toy or comfort item) Trial: Does sleeping with a comfort item make a difference in how well kids sleep compared to not sleeping with a comfort item?

Scientific Title: The Kid's REST (Randomised Evaluation of Sleeping with a Toy or comfort item) Trial: an online, randomised trial of comfort item use on sleep quality in children.

Countries of Recruitment: Recruitment is open globally.

Health Condition(s) or Problem(s) Studied: Sleep-related impairment among primary-school aged children.

Intervention:

Intervention Name: Comfort item group (also called the 'try-it-out' group)

Intervention Description: Participants will choose one comfort item (the comfort item can be any item of choice that a child identifies, for example, a toy, blanket, or other item safe to sleep with) to sleep with for the duration of the trial before starting the trial. For the duration of the trial they will sleep with that same comfort item each night and start using their comfort item when they start getting ready for bed (for example, if they normally read a book before bed, they should use their comfort item then and take it with them when they go to bed). They will sleep in their usual bed each night for the duration of the trial (if a child has more than one home, for example, a boarding school or multiple family homes, both will be considered their usual bed) and keep everything else about their bedtime the same as usual.

Control Name: No treatment group (also called the 'wait-and-see' group)

Control Description: Participants will not sleep with any comfort items at night for the duration of the trial, sleep in their usual bed each night (if a child has more than one home, for example, a boarding school or multiple family homes, both will be considered their usual bed), and keep everything else about their bedtime the same as usual.

<u>Key Inclusion and Exclusion Criteria:</u>
Ages eligible for study: Children, aged between 7 and 12 years
Sexes eligible for study: both
Accepts healthy volunteers: yes

Excludes: Children unable to understand and give assent
Excludes: Children who do not have guardian consent

<u>Study Type:</u> Interventional
<u>Allocation</u>: two-arm randomisation
<u>Intervention model</u>: parallel assignment
<u>Masking</u>: unmasked
<u>Purpose</u>: superiority
<u>Random group allocation:</u> using randomisation feature in the QuestionPro survey software with equal probability (1:1 ratio). Researchers will become aware of allocation post randomisation.

<u>Date of First Enrolment</u>: 13.01.2025

<u>Sample Size:</u> Target number of participants: 292

<u>Recruitment Status:</u> Recruiting

<u>Primary Outcome</u>:
Outcome Name: Sleep-Related Impairment
Metric/method of measurement: Patient-Reported Outcomes Measurement Information System Pediatric Item Bank v1.0-Sleep-Related Impairment – Short Form 4a
Timepoint: Baseline and day 8 post-randomisation

<u>Key Secondary Outcomes:</u>
Secondary Outcome Name: Overall sleep quality
Metric/method of measurement: Single-Item Sleep Quality Scale
Timepoint: Baseline and day 8 post-randomisation

<u>Ethics Review:</u>
Approved, 16 February, 2023

University of Galway Research Ethics Committee: ethics@universityofgalway.ie

Completion Date: 01.06.2025

Summary Results:
Not yet available

IPD Sharing Statement:
The datasets generated during and/or analysed during the current study will be stored in a publicly available repository. The repository used is Open Science Framework (OSF): https://osf.io/tsre8/. The results shared will be those collected from participating kids in the REST trial and will be made available once the trial is complete and after full anonymisation of the data is complete. These records will remain in the OSF database. Parents of participants are aware that data will be published only after it is completely anonymised.
