# Supplementary material for: The REST (randomised evaluation of sleeping with a toy or comfort item) trial: a protocol for an online, randomised trial of comfort item use on sleep quality in children
Source: Contemp Clin Trials Commun. 2025 Nov 25;48:101580. doi: 10.1016/j.conctc.2025.101580 (PMC12702047; doi:10.1016/j.conctc.2025.101580)
Supplement: Supplementary file 4 — Multimedia component 4 [file mmc4.pdf]

The REST (**R**andomised **E**valuation of **S**leeping with a **T**oy or comfort item) Trial Statistical Analysis Plan

Simone Lepage[1, 2], Laura Flight[3], Nikki Totton[4], Declan Devane[1, 2, 5, 6]

1. Health Research Board–Trials Methodology Research Network (HRB-TMRN), University of Galway, Galway, Ireland
2. School of Nursing and Midwifery, University of Galway, Galway, Ireland
3. National Institute for Health and Care Excellence, Piccadilly Plaza, Manchester, United Kingdom
4. Sheffield Centre for Health and Related Research (SCHARR), School of Medicine and Population Health, University of Sheffield, Sheffield, United Kingdom
5. Evidence Synthesis Ireland, University of Galway, Galway, Ireland
6. Cochrane Ireland, University of Galway, Galway, Ireland

Disclosures

The findings and conclusions in this document are those of the authors and not necessarily those of NICE or other authors' employing organisations.

Study title	REST (**R**andomised **E**valuation of **S**leeping with a **T**oy or comfort item) Trial
Study registration number	ISRCTN13756306
Sponsor	Health Research Board-Trials Methodology Research Network of Ireland, College of Medicine, Nursing, and Health Sciences, University of Galway, Ireland
Intervention	Behavioural
Protocol number	V 1.0
Phase	N/A
Statistical analysis plan version	V 1.0
Statistical analysis plan date	25.02.2025
Author	Primary author: Lepage, S.<br>Senior statisticians: Totton, N., Flight, L.<br>Primary supervisor: Devane, D.

Revision history

Version	Revision	Date	Author

List of abbreviations

SAP	Statistical Analysis Plan
RCT	Randomised Controlled Trial
PROMIS	Patient Reported Outcomes Measurement Information System
SRI	Sleep-Related Impairment
SQS	Sleep Quality Scale
CI	Confidence Interval

MID	Minimally Important Difference
SD	Standard Deviation

Introduction

This document outlines the statistical analysis plan (SAP) for the REST trial. This SAP was prepared using the guidance of Stevens et al. [1] and Gamble et al. [2], the associated trial protocol [ref once protocol submitted to HRB Open Research], and trial registration [3].

The objective of this SAP is to outline, a priori, how the aims of the REST trial protocol will be achieved in a statistically rigorous fashion. The SAP will describe which statistical methods, analyses, and data representations will be used to answer the questions the REST trial has set out to answer.

The REST trial is an online, parallel-group, superiority, randomised controlled trial (RCT). The trial's research question emerged from the citizen-science project, The Kid's Trial, where children between 7 and 12 years of age submitted potential research questions, which were then refined through two rounds of voting by the participating children. Further information about the trial's design and planning is detailed and available in the trial protocol [4] and the trial website, www.thekidstrial.ie. Approximately 292 children will be enrolled in the study. The REST trial aims to determine if sleeping with a comfort item (for example, a soft toy or special blanket) makes a difference in kids' sleep compared to not sleeping with a toy or comfort item.

Enrolled children will be randomised to either:

1) the intervention or 'try-it-out' group who will sleep with a comfort item (146 children) or

2) the control or 'wait-and-see' group who will sleep without a comfort item (146 children).

The trial will be carried out at home for seven days. Globally, any child is eligible to enrol if they are between 7 and 12 years of age, can understand English, and can access the trial website: www.thekidstrial.ie. Random group allocation will be carried out with equal probability (1:1 ratio).

Study Methods

Randomisation

The REST trial is a two-arm, parallel group RCT. Random group allocation to either the intervention or the control group will be achieved using the randomisation feature in the QuestionPro Survey Software [5] with equal probability (1:1 ratio). The randomisation feature in QuestionPro uses simple randomisation, and there are no blocks or stratification. We used this platform to maintain consistency with the parent citizen-science project, The Kid's Trial, which used QuestionPro [5] for all survey and data collection processes. Due to resource constraints, researchers will become aware of group allocation after participant randomisation. Because of the nature of the intervention, it is impossible to blind participants to group allocation. No party has any ability to influence allocation.

Outcomes & covariates

The associated Extended Data File (ED.3) accompanying the trial protocol [4] and this SAP contains all instruments and questionnaires.

Primary outcome

- The primary outcome is daytime sleep-related impairment (SRI), which will be measured using the PROMIS (Patient Reported Outcomes Measurement Information System) Pediatric

Short Form v1.0 Sleep-Related Impairment 4a [6] in both the intervention and control groups. Higher scores indicate greater sleep-related impairment.

- Baseline PROMIS SRI measurements will be collected pre-randomisation upon joining the trial (T0).
- Outcome PROMIS SRI measurements will be collected upon trial completion eight days post-randomisation (T1).
- The PROMIS Pediatric Short Form v1.0 Sleep-Related Impairment 4a consists of four questions posed to participants about sleepiness during usual waking hours. The instrument asks the participant to choose one of five categorical answers: Never, Almost Never, Sometimes, Almost Always, Always.
- Each question in the PROMIS Pediatric Short Form v1.0 Sleep-Related Impairment 4a is scored according to the scoring manual, which includes a conversion table to convert each raw score to a T-score [7]. The first three questions receive a raw score of 1 to 5. The last question receives a raw score of 1 to 4 (‘Almost Always’ and ‘Always’ both score as 4 points). Therefore, the instrument's lowest possible total raw score is 4, and the highest possible total raw score is 19.

Secondary Outcomes

- The secondary outcome is sleep quality, measured using the Single-Item Sleep Quality Scale (SQS) [8] in both the intervention and control groups.
- Baseline SQS measurements will be collected pre-randomisation upon joining the trial (T0).
- Outcome SQS measurements will be collected upon trial completion eight days post-randomisation (T1).
- The SQS [8] consists of one question asking participants to rate their overall sleep quality over the previous seven days on a scale of zero to ten on a visual scale, where the numbers increase in units of one. There are five possible answers, which are categorical, where 0 is terrible, 1-3 is poor, 4-6 is fair, 7-9 is good, and 10 is excellent. Therefore, the highest score (excellent or 10) is scored as a 5, and the lowest (terrible or 0) is scored as a 1. No further conversion is needed to score this measure.

Covariates

- Baseline (T0) measurements of SQS and SRI
- Baseline (T0) toy or comfort item use. Usual comfort item use is measured using a custom question, asking participants to describe their normal use of a comfort item on a 3-point Likert scale of Never, Sometimes, Always. This instrument is available in the Extended Data file ED.3.
- Age
- Gender
- Country of residence

Sample size calculation

The sample size calculation is based on a precedent that allows for the use of the repeated measures of the baseline (T0) and post-trial (T1) scores for both the primary and secondary outcome

instruments (PROMIS SRI and SQS) in a linear mixed effects model, using a correlation between the repeated measures of 0.5 [9]. For the primary outcome, PROMIS SRI, we assume a minimally important difference (MID) of a three-point change [10], a standard deviation (SD) of 10 [11], and a dropout rate of 10% [12].

The parameters needed for the calculation:

- MID (minimal important difference) = 3-point change in the PROMIS Paediatric Short Form v.01 Sleep-Related Impairment (SRI) 4a.
- Standard deviation = 10 points.
- Power = 80%
- Significance level = 0.05 (two-sided)
- Pearson's coefficient (r) = 0.5
- Expected dropout rate = 10%.

Sample size calculation (per group) using these parameters:

The base sample size formula for comparing two means is $n = [2\sigma^2 (Z_{\alpha/2} + Z_{\beta})^2 / \delta^2] (1 - r^2)$. The base sample size is 175 participants per group, adjusted for the correlation of repeated measures covariate (r = 0.5), and for a 10% dropout rate, this results in 146 participants per group, with a total of 292 participants.

Interim analyses

The REST trial will not carry out interim analyses. Due to its short duration and low-risk nature, no stopping guidelines are necessary.

Timing of final analysis

Analyses will be carried out collectively when all participating children have reported their results. As per the trial protocol [4], the last time point where results may be collected is 13 days after the final participant has joined and is randomised to their treatment group.

Statistical principles

All data analysis will be carried out using the statistical software R [13]. Categorical variables will be summarised using counts and percentages. Continuous variables will be summarised using mean, median, standard deviation, and range (maximum, minimum) as appropriate. Hypothesis tests will be two-sided, and estimated treatment effects will be accompanied by a 95% confidence interval (CI). All analyses will use a significance level of 0.05.

Adherence and protocol deviations

All participants are asked at T1 whether they followed their group's instructions using custom intervention fidelity questionnaires. The custom questionnaires include Likert scales assessing treatment adherence and vary depending on group allocation. The Likert scales ask participants how often they adhered to their group's instructions and range from Never, 1-2 nights, 3-4 nights, 5-6 nights, and Always. If the participant answers 3-4 nights or less frequently, they are considered non-adherent and are asked why they deviated from their group's instructions. The custom questionnaires are available in Extended Data ED.3.

The REST trial, including enrolment, occurs completely online. Therefore, a participant may enrol in the trial more than once, for example, if they are unhappy with their treatment group allocation or by mistake. These instances are referred to as duplicate enrolments. Enrolment data will be reviewed daily during the trial recruitment period, and in the case of a duplicate enrolment, we will

address them as follows. A participant who has completed the entire enrolment survey in QuestionPro [5] and been allocated to a group more than once may have received the same group allocation for each enrolment, or they may not have, as allocation is random. In this case, an email is sent to the participant's guardian clarifying that their first enrolment should be considered their 'correct' group allocation, and we will be collecting their results based on this allocation. The duplicate enrolment is not considered in terms of sample size recruitment and is 'suspended'.

Analysis population

The primary analysis population is an Intention-To-Treat (ITT) approach where all participants randomised in the REST trial will be treated as the analysis population, regardless of completion status, the exception to this is duplicate enrolment as described above. A secondary population analysis, the Per-Protocol population (PP), where participants who were adherent to their treatment group will be carried out using the definition described above.

Trial population

Inclusion and exclusion criteria

All participants are self-referred. Participants are primary school-aged children (7-12 years old) who meet the inclusion criteria. The REST trial, is open to any child around the world, so there are no geographical exclusion criteria; however, due to budgetary constraints, we could not translate all trial materials into other languages, so a good understanding of English is needed. Inclusion and exclusion criteria are listed below. Further details are outlined in the trial protocol [4].

Inclusion Criteria:

- Children aged 7 to 12 years.
- Proficiency in English sufficient to understand trial materials.
- Access to the trial's online platform.
- Guardian consent for participation.

Exclusion Criteria:

- Inability to understand and provide assent.

Missing data

All reported outcome measures, PROMIS SRI and SQS, are collected via a participant's guardian using a link that directs them to the survey accompanying their allocated group. If the survey is completed, there can be no missing data as the survey does not allow a participant to 'skip' any of the questions. Participants who do not complete the survey at T1 collecting the results of their trial will be considered missing data. Therefore, no missing data mechanisms will be used. However, if overall missing data exceeds 10% for the primary outcome, sensitivity analyses using multiple imputation will be performed to assess the robustness of the primary analysis.

Statistical analysis

Baseline data

At baseline (T0), demographic information, including age, gender, ethnicity, and country of residence, will be collected alongside baseline measurements of usual comfort item use, PROMIS SRI and SQS. Categorical variables will be summarised using counts and percentages. Continuous variables will be summarised using mean, median, standard deviation, and range (maximum, minimum). Summary statistics will be presented for each treatment group and overall.

Primary and secondary analyses

With two time-point measurements (T0 and T1) for both outcomes (SIR, SQS), we will use a mixed effects model effect to analyse our data. Mixed effects models are increasingly used to analyse participant-reported outcomes (PROs) compared to historically more frequently used models for PROs, such as simple t-tests and linear regression models [14]. In our analysis, the treatment group serves as a predictor, and the fixed covariates are baseline measurements of the outcome being analysed, baseline comfort item use, age, and gender. The country of residence will serve as the random effect. The intervention effect will be estimated using the inclusion of the treatment group as a predictor. Model assumptions will be checked using standard methods from the residuals. If any violations are noted, we will address them with a sensitivity analysis. Secondary analyses will be handled in the same manner as primary analyses.

Subgroup analyses

Exploratory subgroup analyses will be conducted to examine whether the treatment effect varies by:

- Baseline comfort item use (Never vs. Sometimes vs. Always)
- Age groups (7-9 years vs. 10-12 years)
- Gender

These analyses will be performed by including interaction terms between the treatment and the subgroup variable in the primary analysis model.

Sensitivity analyses

The following sensitivity analyses will be conducted to assess the robustness of the primary analysis results:

1. Per-Protocol analysis including only participants who adhered to their allocated intervention for at least 5 nights during the trial.
2. If applicable, analysis with multiple imputation for missing outcome data if the missing data rate exceeds 10%.

Harms

We do not anticipate any emotional or physical risks or disturbances to participants or their guardians in the REST trial. For further information about possible harms, please refer to the trial protocol document [4].

References

1. Stevens, G., et al., *A template for the authoring of statistical analysis plans.* Contemporary Clinical Trials Communications, 2023. **34**: p. 101100.
2. Gamble, C., et al., *Guidelines for the Content of Statistical Analysis Plans in Clinical Trials.* JAMA, 2017. **318**(23): p. 2337-2343.
3. ISRCTN, *Does sleeping with a comfort item make a difference in how well kids sleep compared to not sleeping with a comfort item?* 2025, ISRCTN: online.
4. Lepage, S., Flight, L., Totton, N., Devane, D., *The REST (Randomised Evaluation of Sleeping with a Toy or comfort item): a protocol for an online, randomised trial of comfort item use on sleep quality in children.* 2025, School of Nursing and Midwifery, University of Galway.
5. QuestionPro, *QuestionPro Survey Software*. 2025.
6. Bevans, K.B., et al., *Qualitative Development and Content Validation of the PROMIS Pediatric Sleep Health Items.* Behav Sleep Med, 2019. **17**(5): p. 657-671.

7. Carle, A.C., et al., *Using nationally representative percentiles to interpret PROMIS pediatric measures.* Qual Life Res, 2021. **30**(4): p. 997-1004.
8. Snyder, E., et al., *A New Single-Item Sleep Quality Scale: Results of Psychometric Evaluation in Patients With Chronic Primary Insomnia and Depression.* J Clin Sleep Med, 2018. **14**(11): p. 1849-1857.
9. Walters, S.J., et al., *Sample size estimation for randomised controlled trials with repeated assessment of patient-reported outcomes: what correlation between baseline and follow-up outcomes should we assume?* Trials, 2019. **20**(1).
10. Thissen, D., et al., *Estimating minimally important difference (MID) in PROMIS pediatric measures using the scale-judgment method.* Qual Life Res, 2016. **25**(1): p. 13-23.
11. Forrest, C.B., et al., *Development and validation of the PROMIS Pediatric Sleep Disturbance and Sleep-Related Impairment item banks.* Sleep, 2018. **41**(6).
12. Yu, S.H., et al., *Addressing the Challenges of Recruitment and Retention in Sleep and Circadian Clinical Trials.* Behavioral Sleep Medicine, 2020. **18**(1): p. 23-34.
13. R, *A Language and Environment for Statistical Computing*, R.C. Team, Editor. 2023, R: Foundation for Statistical Computing Vienna, Austria.
14. Qian, Y., et al., *Comprehensive review of statistical methods for analysing patient-reported outcomes (PROs) used as primary outcomes in randomised controlled trials (RCTs) published by the UK's Health Technology Assessment (HTA) journal (1997–2020).* BMJ Open, 2021. **11**(9): p. e051673.
