# Supplementary material for: The REST (randomised evaluation of sleeping with a toy or comfort item) trial: a protocol for an online, randomised trial of comfort item use on sleep quality in children
Source: Contemp Clin Trials Commun. 2025 Nov 25;48:101580. doi: 10.1016/j.conctc.2025.101580 (PMC12702047; doi:10.1016/j.conctc.2025.101580)
Supplement: Supplementary file 5 — Multimedia component 5 [file mmc5.pdf]

Day 8 Post-randomisation sleep questions: Control

QuestionPro

It's time for you to tell us how your trial went!

Remember, it's important to tell us what you really did. This helps us understand the true results of the trial.

* In the last 7 nights, how often did you:

If you're on a mobile phone, move the button to choose from: Never, 1-2 nights, 3-4 nights, 5-6 nights, Always

Sleep with a comfort item?

Sleep in your usual bed?

*** Why do you think sleeping WITHOUT a comfort item was tricky?**

- I sometimes forgot and used one.
- I really wanted to use a comfort item.
- I'm not sure.
- Another reason you can write here

*** Why did you sleep somewhere other than your usual bed?**

- I had to travel or spend time away from my usual bed.

- ◯ I'm not sure.
- ◯ Another reason you can write here

*** Before the trial, did you usually sleep with a comfort item?**

- ◯ Never
- ◯ Sometimes
- ◯ Always

Now for the fun part- How was your sleep!

* Over the past 7 days:

If you're on a **mobile phone**, move the button to choose from: Never, Almost never, Sometimes, Almost always, Always

I was sleepy during the daytime.

I had a hard time concentrating because I was sleepy.

I had a hard time getting things done because I was sleepy.

I had problems during the day because of poor sleep.

QuestionPro

* **Over the past 7 days, how would you rate your sleep?**

This means how well you slept over the past week. Think about how much sleep you got, how easy it was to fall asleep, and if you woke up often at night.

	Terrible (0)	Poor (1, 2, 3)	Fair (this means ok) (4, 5, 6)	Good (7, 8, 9)	Excellent (10)
**Overall sleep**	◯	◯	◯	◯	◯

* **If you usually sleep with a comfort item, how did you feel about not using one during the trial?**

◯ It was very tricky.

◯ It was a little tricky.

◯ It didn't bother me.

◯ I liked not using one.

◯ This doesn't apply to me because I don't usually use a comfort item.
