# Supplementary material for: The REST (randomised evaluation of sleeping with a toy or comfort item) trial: a protocol for an online, randomised trial of comfort item use on sleep quality in children
Source: Contemp Clin Trials Commun. 2025 Nov 25;48:101580. doi: 10.1016/j.conctc.2025.101580 (PMC12702047; doi:10.1016/j.conctc.2025.101580)
Supplement: Supplementary file 6 — Multimedia component 6 [file mmc6.pdf]

Day 8 Post-randomisation sleep questions: Intervention

It's time for you to tell us how your trial went!

Remember, it's important to tell us what you really did. This helps us understand the true results of the trial.

* **Over the last 7 nights, how often did you:**

If you're on a **mobile phone,** move the button to choose from: Never, 1-2 nights, 3-4 nights, 5-6 nights, Always

Sleep with a comfort item each night?

Use the same comfort item each night?

Start using your comfort item when you got ready for bed each night.

Sleep in your usual bed each night.

* **Why do you think sleeping WITH a comfort item was tricky?**

- ○ I sometimes forgot.
- ○ I couldn't find an item I liked.
- ○ I'm not sure.

- ◯ Another reason you can write here.

*** Why do you think sleeping with the SAME comfort item was tricky?**

- ◯ I sometimes forgot.
- ◯ I didn't like the first item I chose.
- ◯ I'm not sure.
- ◯ Another reason you can write here.

You can answer below.

*** Why do you think it was tricky to start using your comfort item when you got ready for bed each night?**

- ◯ I sometimes forgot.
- ◯ I'm not sure.
- ◯ Another reason you can write here

*** Why did you sleep somewhere other than your usual bed?**

- ◯ I had to travel or spend time away from my usual bed.

QuestionPro

◯ I'm not sure.

◯ Another reason you can write here

* **Before the trial, did you usually sleep with a comfort item?**

◯ Never

◯ Sometimes

◯ Always

Now for the fun part- How was your sleep?

* **Over the past 7 days:**

If you're on a **mobile phone**, move the button to choose from: Never, Almost never, Sometimes, Almost always, Always

I was sleepy during the daytime.

I had a hard time concentrating because I was sleepy.

I had a hard time getting things done because I was sleepy.

I had problems during the day because of poor sleep.

*** Over the past 7 days, how would you rate your sleep?**

This means how well you slept over the past week. Think about how much sleep you got, how easy it was to fall asleep, and if you woke up often at night.

	Terrible (0)	Poor (1, 2, 3)	Fair (this means ok) (4, 5, 6)	Good (7, 8, 9)	Excellent (10)
**Overall sleep**	◯	◯	◯	◯	◯

*** If you don't usually sleep with a comfort item, how did you feel about using one during the trial?**

◯ It was very tricky.

◯ It was a little tricky.

◯ It didn't bother me.

◯ I liked using one.

◯ This doesn't apply to me because I usually use a comfort item.
