# Supplementary material for: The REST (randomised evaluation of sleeping with a toy or comfort item) trial: a protocol for an online, randomised trial of comfort item use on sleep quality in children
Source: Contemp Clin Trials Commun. 2025 Nov 25;48:101580. doi: 10.1016/j.conctc.2025.101580 (PMC12702047; doi:10.1016/j.conctc.2025.101580)
Supplement: Supplementary file 7 — Multimedia component 7 [file mmc7.pdf]

Baseline sleep questions

QuestionPro

This is the exciting bit - It's time for you to join The Kid's Trial!

Once you know your group, it's important to follow the instructions for all 7 days even if it feels different from your usual routine.

Remember, no one knows what group you'll be in, so when you agree to join the trial, be ready to either:

- sleep WITH a comfort item (for example, a soft toy or special blanket, or any other item you choose) for the next 7 nights.

OR

- NOT to sleep with any comfort item for the next 7 nights.

Before we start, let's see how well you think you usually sleep.

* **Over the past 7 days:**

If you're on a **mobile phone**, move the button to choose from: Never, Almost never, Sometimes, Almost always, Always

I was sleepy during the daytime.

I had a hard time concentrating because I was sleepy.

I had a hard time getting things done because I was sleepy.

I had problems during the day because of poor sleep.

* Over the past 7 days, how would you rate your sleep?

This means how well you slept over the past week. Think about how much sleep you got, how easy it was to fall asleep, and if you woke up often at night.

	Terrible (0)	Poor (1, 2, 3)	Fair (this means ok) (4, 5, 6)	Good (7, 8, 9)	Excellent (10)
Overall sleep	○	○	○	○	○

*** Do you usually sleep with a comfort item?**

○ Never

○ Sometimes

○ Always

QuestionPro
